# Supplementary material for: Modeling glioblastoma heterogeneity as a dynamic network of cell states
Source: Mol Syst Biol. 2021 Sep 16;17(9):e10105. doi: 10.15252/msb.202010105 (PMC8444284; doi:10.15252/msb.202010105)
Supplement: Supplementary file 6 — Source Data for Figure 5 [file MSB-17-e10105-s004.zip › Figure5A_sourcedata/GSEA_3071/hallmarks_stateVI.GseaPreranked.1623742557031/HALLMARK_KRAS_SIGNALING_DN.html]

Details for gene set HALLMARK\_KRAS\_SIGNALING\_DN[GSEA]

|  || Dataset | state63071 |
| Phenotype | NoPhenotypeAvailable |
| Upregulated in class | na\_neg |
| GeneSet | HALLMARK\_KRAS\_SIGNALING\_DN |
| Enrichment Score (ES) | -0.52818775 |
| Normalized Enrichment Score (NES) | -1.9450674 |
| Nominal p-value | 0.004201681 |
| FDR q-value | 0.023378186 |
| FWER p-Value | 0.075 |
Table: GSEA Results Summary

  

Fig 1: Enrichment plot: HALLMARK\_KRAS\_SIGNALING\_DN      
 Profile of the Running ES Score & Positions of GeneSet Members on the Rank Ordered List

  

| PROBE | GENE SYMBOL | GENE\_TITLE | RANK IN GENE LIST | RANK METRIC SCORE | RUNNING ES | CORE ENRICHMENT || 1 | EDN1 |  |  | 50 | 0.866 | 0.0373 | No |
| 2 | COPZ2 |  |  | 457 | 0.469 | -0.0474 | No |
| 3 | NUDT11 |  |  | 1028 | 0.340 | -0.1850 | No |
| 4 | PLAG1 |  |  | 1089 | 0.333 | -0.1820 | No |
| 5 | IFI44L |  |  | 1742 | 0.256 | -0.3473 | No |
| 6 | GPRC5C |  |  | 1976 | -0.274 | -0.3956 | No |
| 7 | SNN |  |  | 2012 | -0.281 | -0.3887 | No |
| 8 | CLSTN3 |  |  | 2202 | -0.314 | -0.4224 | No |
| 9 | SLC25A23 |  |  | 2323 | -0.337 | -0.4358 | No |
| 10 | DCC |  |  | 2658 | -0.414 | -0.5038 | Yes |
| 11 | EFHD1 |  |  | 2686 | -0.421 | -0.4864 | Yes |
| 12 | SLC16A7 |  |  | 2745 | -0.438 | -0.4766 | Yes |
| 13 | CNTFR |  |  | 2748 | -0.440 | -0.4512 | Yes |
| 14 | YPEL1 |  |  | 2781 | -0.449 | -0.4335 | Yes |
| 15 | GAMT |  |  | 2915 | -0.500 | -0.4408 | Yes |
| 16 | CELSR2 |  |  | 3121 | -0.598 | -0.4622 | Yes |
| 17 | PTPRJ |  |  | 3174 | -0.640 | -0.4388 | Yes |
| 18 | SKIL |  |  | 3188 | -0.652 | -0.4039 | Yes |
| 19 | BARD1 |  |  | 3277 | -0.736 | -0.3848 | Yes |
| 20 | TGFB2 |  |  | 3340 | -0.804 | -0.3545 | Yes |
| 21 | TFAP2B |  |  | 3343 | -0.807 | -0.3074 | Yes |
| 22 | BTG2 |  |  | 3377 | -0.857 | -0.2660 | Yes |
| 23 | KCNQ2 |  |  | 3471 | -1.051 | -0.2297 | Yes |
| 24 | KLHDC8A |  |  | 3552 | -1.370 | -0.1710 | Yes |
| 25 | IGFBP2 |  |  | 3634 | -3.295 | 0.0011 | Yes |
Table: GSEA details [plain text format]

  

Fig 2: HALLMARK\_KRAS\_SIGNALING\_DN: Random ES distribution      
 Gene set null distribution of ES for **HALLMARK\_KRAS\_SIGNALING\_DN**
